# Comorbidities are associated with poorer quality of life and functioning and worse symptoms in the 5 years following colorectal cancer surgery: Results from the ColoREctal Well‐being (CREW) cohort study

**DOI:** 10.1002/pon.4845

**Published:** 2018-09-13

**Authors:** Amanda Cummings, Chloe Grimmett, Lynn Calman, Mubarak Patel, Natalia Vadimovna Permyakova, Jane Winter, Jessica Corner, Amy Din, Deborah Fenlon, Alison Richardson, Peter W. Smith, Members of CREW Study Advisory Committee, Claire Foster

**Affiliations:** ^1^ Macmillan Survivorship Research Group, Health Sciences University of Southampton Southampton UK; ^2^ Social Statistics and Demography, Social Sciences University of Southampton Southampton UK; ^3^ University Hospital Southampton NHS Foundation Trust Southampton UK; ^4^ Executive Office University of Nottingham Nottingham UK; ^5^ College of Health and Human Sciences Swansea University Swansea UK; ^6^ Health Sciences University of Southampton Southampton UK

**Keywords:** cancer, colorectal cancer, comorbidities, health and well‐being, longitudinal, oncology, quality of life, survivorship

## Abstract

**Objective:**

More people are living with the consequences of cancer and comorbidity. We describe frequencies of comorbidities in a colorectal cancer cohort and associations with health and well‐being outcomes up to 5 years following surgery.

**Methods:**

Prospective cohort study of 872 colorectal cancer patients recruited 2010 to 2012 from 29 UK centres, awaiting curative intent surgery. Questionnaires administered at baseline (pre‐surgery), 3, 9, 15, 24 months, and annually up to 5 years. Comorbidities (and whether they limit activities) were self‐reported by participants from 3 months. The EORTC QLQ‐C30 and QLQ‐CR29 assessed global health/quality of life (QoL), symptoms, and functioning. Longitudinal analyses investigated associations between comorbidities and health and well‐being outcomes.

**Results:**

At baseline, the mean age of participants was 68 years, with 60% male and 65% colon cancer. Thirty‐two per cent had 1 and 40% had ≥2 comorbidities. The most common comorbidities were high blood pressure (43%), arthritis/rheumatism (32%), and anxiety/depression (18%). Of those with comorbidities, 37% reported at least 1 that limited their daily activities. Reporting any limiting comorbidities was associated with poorer global health/QoL, worse symptoms, and poorer functioning on all domains over 5‐year follow‐up. Controlling for the most common individual comorbidities, depression/anxiety had the greatest deleterious effect on outcomes.

**Conclusions:**

Clinical assessment should prioritise patient‐reported comorbidities and whether these comorbidities limit daily activities, as important determinants of recovery of QoL, symptoms, and functioning following colorectal cancer. Targeted interventions and support services, including multiprofessional management and tailored assessment and follow‐up, may aid recovery of health and well‐being in these individuals.

## 1 BACKGROUND

Colorectal cancer (CRC) is one of the most common cancers worldwide, with an estimated incidence of over 1.3 million, and this is increasing.^1^ Five‐year survival rates in the UK stand at 57% and 65% in the United States.^2^, ^3^ Colorectal cancer is more likely in older adults, with 60% of survivors aged over 65 years.^4^

Comorbidity is defined as the presence of distinct medical condition(s) in addition to the particular index disease, in this case CRC.^5^ Multiple comorbidity is progressively more common with age; thus, older CRC survivors generally present with high levels of comorbidity.^6^ Colorectal cancer survivors also have higher rates of comorbid disease compared with the general population,^7^ with around 40% to 50% of CRC patients reported to have ≥2 comorbidities.^8^, ^9^

Living with comorbidity after CRC diagnosis is now the norm rather than the exception. Therefore, investigation into how comorbidities affect CRC survivors' health and well‐being has become increasingly important. Cancer survivors often report poorer health and well‐being compared to healthy populations, and independently, long‐term chronic conditions negatively influence quality of life (QoL).^10^, ^11^

Whilst there is a growing body of literature exploring the effect of comorbidities in people recovering from CRC, there is significant variability in study sample sizes,^12^, ^13^ participant characteristics,^14^, ^15^ and time points of assessment,^9^, ^12^ and it is not always possible to identify CRC‐specific data in cohort studies that include multiple tumour groups.^16^ In addition, investigations of the impact of comorbidities on QoL, symptoms, and functioning following a CRC diagnosis are limited by cross‐sectional design,^9^, ^17^ a narrow range of outcomes,^17^, ^18^ and methods used to determine comorbidity status.^17^, ^19^

Most studies focus on the *number* of comorbidities,^9^, ^20^ or comorbidity *severity* using weighted scales, where severity is based on the predefined mortality risk of individual conditions, such as the Charlson Comorbidity Index^13^, ^19^, ^21^ or similar indices.^22^ Few studies describe patient‐reported severity, such as limitations on activities caused by comorbidities. Those that do are either cross‐sectional, limited to self‐reported depression, do not exclusively examine the impact of comorbidity limitation on well‐being, or present data from mixed tumour groups.^12^, ^19^, ^23^

Few studies have described associations between comorbidities, and health and well‐being over time. Associations with pain, fatigue, and mental well‐being up to 1 year following a CRC diagnosis, and fatigue and QoL over time in longer term survivors, have been described, yet only in relation to the number of comorbidities.^18^, ^24^ The role of individual comorbid conditions is largely overlooked in studies.

Only one longitudinal study has mapped comorbidity prevalence up to 1 year; however, this study was nonpopulation based and limited to CRC survivors >65 years.^14^ Similarly, no studies describe the demographic, clinical, and treatment characteristics of CRC survivors with comorbidities. Using results from the ColoREctal Well‐being (CREW) study,^25^ a longitudinal cohort study investigating recovery of health and well‐being in the 5 years following colorectal cancer, this paper aims to determine the following:

1. The frequency of comorbidities, their limiting effects on daily activities, and the frequency of individual comorbid conditions among CRC survivors.
2. The association between comorbidities, and recovery of QoL, symptom, and functioning outcomes.
3. The demographic and clinical factors that characterise comorbid CRC survivors.

## 2 METHODS

### 2.1 Design

The ColoREctal Well‐being (CREW) study is a prospective longitudinal cohort study of patients with nonmetastatic CRC undergoing curative intent surgery. Further details are described elsewhere.^25^

### 2.2 Participants

Eligible patients had a diagnosis of Dukes' stage A‐C colorectal cancer, were being treated with curative intent surgery, aged ≥18 years, and able to complete questionnaires. Having a prior cancer diagnosis was an exclusion criterion.

### 2.3 Procedure

Participants were recruited from 29 UK hospitals between November 2010 and March 2012. Self‐report questionnaires were completed before surgery (baseline), and mailed questionnaires were sent at regular intervals: 3, 9, 15, 24 months, and annually up to 5 years. Clinical and treatment characteristics were identified from NHS medical data. Ethical approval was granted by the UK NHS Health Research Authority NRES Committee South Central—Oxford B (REC ref: 10/H0605/31).

### 2.4 Measures

Full details of the measures used in CREW have been published.^25^ Measures that pertain to the findings presented in this paper are summarised below.

#### 2.4.1 Socio‐demographic, clinical, and treatment data

Clinical and treatment data were obtained (with consent) from medical notes: tumour site, Dukes' stage, nodal involvement, how CRC was detected, family history of CRC, presence of a stoma, and neoadjuvant and adjuvant treatment. Neighbourhood deprivation was derived from postcodes using the index of multiple deprivation.^26^ Domestic and employment status were assessed by participant self‐report in questionnaires.

#### 2.4.2 Comorbidity data

**Figure 1:**
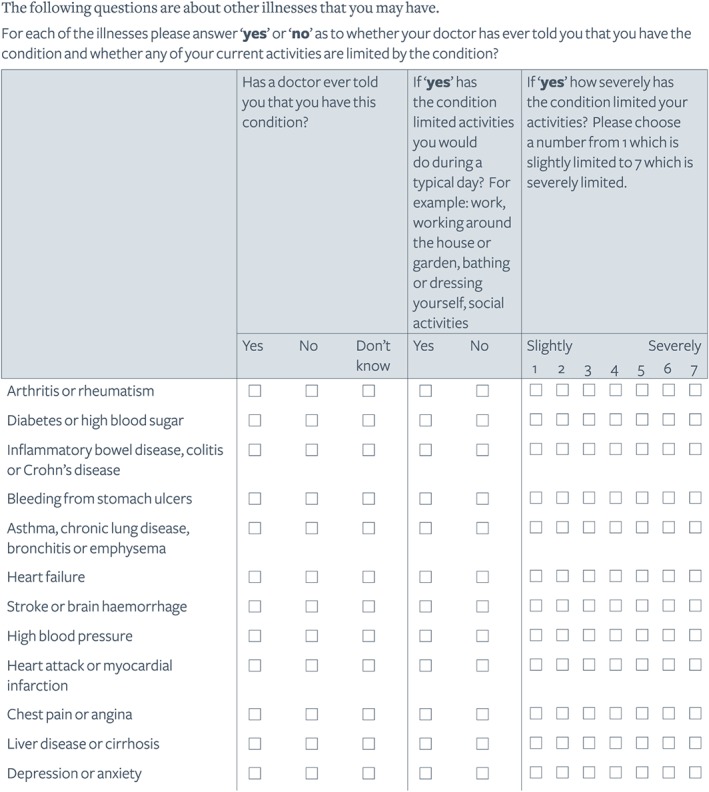
Self‐report comorbidity section of CREW questionnaires

Patient self‐reported comorbidity status remains an accurate method for health research against clinical record review.^27^ Self‐reported comorbidity data were collected at 3, 15, 24, 36, 48, and 60 months. The list relating to 12 individual conditions or disease groups was a study‐specific measure (not formally validated) informed by Ramsey et al,^12^ with format informed by the Self‐Administered Comorbidity Questionnaire.^28^ The list (Figure 1) asks whether a doctor has ever told the participant they have the condition, whether the condition limits typical daily activities, and the severity of such impact (ranked from 1 to 7 on Likert scale). At 24 months, an additional question asked whether each condition had been diagnosed before or after CRC diagnosis.

#### 2.4.3 QoL, symptoms, and functioning

Quality of life, symptoms, and functioning were assessed using the validated European Organisation for Research and Treatment of Cancer QoL (EORTC QLQ) core (C30) questionnaire^29^ and the CRC component (CR29),^30^ from 3 months onwards.

Global health status/QoL scale of the QLQ‐C30 was used to represent overall QoL (comprises 2 items). Analyses of symptoms focussed on those most frequently reported in CRC^9^, ^13^, ^31^: pain, fatigue (from QLQ‐C30), urinary, and bowel symptoms (from QLQ‐CR29). Physical, role, cognitive, emotional, and social functioning was assessed using QLQ‐C30 subscales.

### 2.5 Statistical analysis

Subscale scores from the EORTC questionnaires were calculated according to published guidelines.^29^ To avoid problems with multiple testing of a large number of individual symptoms, summary scores representing urinary symptoms and bowel symptoms were calculated by taking the mean of QLQ‐CR29 subscales: (a) urinary frequency, urinary incontinence, and dysuria for urinary symptoms and (b) blood and mucus in stool, stool frequency, abdominal pain, pain in buttocks/anal area/rectum, bloating, flatulence, and faecal incontinence for bowel symptoms.

Because of initial analyses indicating the stability in prevalence and chronicity of comorbidities over follow‐up, statistical analyses used 3‐month comorbidity data.

In the first part of the analyses, associations between the number of comorbidities reported at 3 months and baseline socio‐demographic, clinical, and treatment factors were assessed using the chi‐square test or chi‐square test for trend, where appropriate. The index of multiple deprivation was categorised into quintiles.^26^ Performance status was not captured.

For the second part, longitudinal analyses were conducted using generalised estimating equations, based on all available completed questionnaires up to 60 months. Analyses assessed the associations between EORTC subscale scores as dependent variables and comorbidities reported at 3 months (5 most prevalent comorbid conditions and the comorbidity status itself categorised as none, nonlimiting, or limiting) as independent variables at the 5% significance level. Separate models were fitted for each EORTC subscale of global health status/QoL, symptoms, and functioning.

The first set of multivariable regression models included independent variables separately in each model and was adjusted for time since surgery and those demographic, clinical, or treatment factors significantly associated with total numbers of comorbidities in the first analyses.

The second set of multivariable regression models focused on examining multiple effects of the significant comorbidity predictors. Independent (comorbidity) variables statistically significant in the first set of regression models were put together in the second set, again adjusting for time since surgery and demographic/clinical/treatment factors identified as significant in initial analyses.

Participants with missing questionnaires were included in analyses for time points for which they provided data; there was no imputation of missing questionnaires, or socio‐demographic, clinical, treatment, or comorbidity data. Missing data on the EORTC measures were dealt with using published guidelines.^29^, ^30^

Longitudinal analyses involving individual comorbidities encompassed the 5 most prevalent individual conditions (small numbers restricted detailed analysis for less prevalent comorbidities and any associations of individual conditions that limited daily activities).

In line with published guidance, clinically meaningful differences were determined by a >10‐point difference in EORTC subscale scores.^32^

## 3 RESULTS

### 3.1 Participants

One thousand three hundred and fifty eligible individuals were identified. Of those eligible, 78% (n = 1055) agreed to participate, of whom 86% (n = 909) gave full consent to participate and 14% (n = 146) gave permission for only clinical data to be collected. Thirty‐seven were found to be ineligible following surgery. Excluding 11 individuals who withdrew or died between consent and baseline, 861 participants consented to follow‐up. This sample is representative of eligible patients treated during the recruitment period. Response rates were 88% at baseline and 69% at 60 months. Comorbidity data were available for 99% of those responding (n = 659) at 3 months and 87% (n = 324) at 60 months.

Mean age at baseline was 68 years (range 27 to 95 years). The majority were of white ethnic origin, and 60% were male. Most participants were retired (60%), and over 60% were married or living with a partner. Most participants had colon cancer (65%), 35% rectal tumours. Over 53% had Dukes' stage B, 20% had stage C1, and 12% to 14% had stage A or C2 (1% was undetermined), Eighteen per cent received neoadjuvant treatment and 46% adjuvant chemotherapy or radiotherapy.

### 3.2 Frequency and impact of comorbidities

At 3 months, 28% reported no comorbidities, 32% reported 1, 23% 2, and 17% 3 or more. Of the 72% (n = 476) with comorbidities, the median number was 2. Of those with comorbidities, 37% reported at least 1 that limited their daily activities, with 13% reporting 2 or more limiting comorbidities (Table 1). The proportion of limiting comorbidities remained consistent over time. Most participants (62% at 3 months) reported that their comorbidities limited daily activities “moderately”, which remained fairly consistent over follow‐up (Appendix 1).

**Table 1:** Number of comorbidities and number of limiting comorbidities for the CREW cohort (reported at 3 months following primary CRC surgery)

Number of Comorbidities	3 months n = 659	Number of Limiting Comorbidities	3 months n = 476
0	183 (27.7%)	0	249 (52.3%)
1	211 (32.0%)	1	115 (24.2%)
2	150 (22.8%)	≥2	62 (13.0%)
≥3	115 (17.4%)	Missing data	50 (10.5%)
Presence of any comorbidities	476 (72.2%)	Presence of any limiting comorbidities	177 (37.2%)

### 3.3 Individual comorbidities

The most common individual comorbidities reported at 3 months were high blood pressure (43%), arthritis/rheumatism (arthritis) (32%), depression/anxiety (18%), diabetes/high blood sugar (diabetes) (16%), and asthma/chronic lung disease (lung disease) (16%). There was less than a 7% change in the prevalence of all conditions over follow‐up (Appendix 2).

Results suggest that the majority of comorbid conditions were diagnosed prior to CRC diagnosis (participants responded to this question at 24 months). The exceptions to this were stroke/brain haemorrhage and liver disease/cirrhosis, of which 50% and 80% (respectively) were diagnosed following CRC diagnosis. Of note is the relatively high percentage (46%) of comorbid depression/anxiety diagnosed post CRC diagnosis, although numbers were small for analysis. All other conditions (apart from inflammatory bowel disease) were diagnosed before CRC diagnosis in >78% of individuals.

Arthritis and heart failure were reported to be the most limiting conditions. Of participants reporting these conditions, >50% stated it limited their daily activities. Stroke/brain haemorrhage, myocardial infarction, and angina were reported as limiting by ≥40% of respondents with each condition, and >35% of participants with depression/anxiety and lung disease reported them as limiting. High blood pressure was the most prevalent, but least limiting condition. Of participants with diabetes, 14% reported the condition as limiting (Appendix 2).

### 3.4 Demographic and clinical characteristics

Socio‐demographic, clinical, and treatment characteristics of CRC patients and their associations with comorbidities are presented in Appendix 3. Ethnicity is not presented as numbers in minority groups were too small for analysis. Comorbidities were significantly more common in older, retired, or unemployed respondents. No significant associations were found between comorbidities and any other socio‐demographic, clinical, or treatment factors, nor for comorbidities that limited daily activities.

### 3.5 Comorbidities and QoL, symptom, and functioning outcomes

Because of high correlation between age and employment status, only age at baseline was included in the multivariable regression analyses.

The first set of longitudinal multivariable regression models adjusted for age and time since surgery (from baseline to 60 months) illustrates that the presence of any limiting comorbidities was significantly associated with poorer global health status/QoL, symptom, and functioning outcomes across all domains (*P* < .001), including increased fatigue, pain, urinary, and bowel symptoms and reduced physical, role, emotional, cognitive, and social functioning (Appendix 4). Findings illustrated clinically meaningful differences associated with the presence of limiting comorbidities across all outcomes (except for urinary and bowel symptoms). In contrast, the presence of nonlimiting comorbidities was only significantly associated with increased pain and worse physical functioning (*P* < .05).

Of the 5 most prevalent individual comorbid conditions reported at 3 months, arthritis and depression/anxiety were significantly associated with poorer global health status/QoL, symptom, and functioning outcomes across all domains (*P* < .001). Depression/anxiety appeared to have the greatest association with poorer outcomes, with clinically meaningful differences across all outcomes (except for urinary and bowel symptoms). Lung disease was also significantly associated with poorer outcomes, with the exception of urinary symptoms. Diabetes and high blood pressure were significantly associated with increased pain and poorer physical functioning, with diabetes also associated with worse urinary symptoms (Appendix 4).

Once adjusted for all significant comorbidity predictors, final multivariable regression models confirmed that the presence of any limiting comorbidities remained a statistically strong and significant predictor of all health and well‐being outcomes (*P* < .001), with the exception of emotional functioning (Table 2). The biggest and clinically significant differences were observed for pain, fatigue, physical, role, social, and cognitive functioning.

**Table 2:** Mean differences in EORTC subscale scores over follow‐up between 3 and 60 months following surgery, estimated from multivariable regression models adjusted for age at baseline, time since surgery, comorbidity status, and 5 most prevalent conditions

Independent Variables	Dependent Variables: EORTC Subscale Scores^a^									
	Global Health Status/QoL^b^	Fatigue^c^	Pain^c^	Urinary Symptoms^c^^,^ d	Bowel Symptoms^c^^,^ e	Physical Functioning^b^	Role Functioning^b^	Emotional Functioning^b^	Cognitive Functioning^b^	Social Functioning^b^
	Model 1	Model 2	Model 3	Model 4	Model 5	Model 6	Model 7	Model 8	Model 9	Model 10
1) Comorbidity status*:*										
None (ref)	0	0	0	0	0	0	0	0	0	0
Yes, nonlimiting comorbidities	0.4	0.4	2.8	0.6	1.4	−1.7	0.2	1.3	−0.7	0.6
Yes, limiting comorbidities	−8.3***	13.6***	19.1***	6.3***	6.4***	−16.3***	−15.0***	−5.1	−10.2***	−11.3***
2) High blood pressure										
No (ref)			0			0				
Yes			−1.0			0.01				
3) Arthritis/rheumatism										
No (ref)	0	0	0	0	0	0	0	0	0	0
Yes	−3.2	3.3	4.8	0.9	0.9	−0.9	−3.2	−0.9	0.5	−3.6
4) Depression/anxiety										
No (ref)	0	0	0	0	0	0	0	0	0	0
Yes	−10.0***	12.6***	6.7*	3.2	4.2*	−8.7**	−9.1**	−18.8***	−9.6**	−10.4***
5) Diabetes/high blood sugar										
No (ref)			0	0		0				
Yes			1.6	2.7		−2.2				
6) Asthma/chronic lung disease										
No (ref)	0	0	0		0	0	0	0	0	0
Yes	−5.2*	3.0	−0.3		−0.3	−5.0*	−4.5	−1.8	−1.8	0.2
7) Age at baseline	−0.1	0.01	−0.2	0.1*	−0.3***	−0.4***	−0.1	0.2*	0.1	0.1
8) Time since surgery	0.1***	−0.2***	−0.1**	0.02	−0.1***	0.1**	0.2***	0.1***	0.2***	0.3***

Grey areas indicate that the referred independent variable was not included in the final multivariable model of the referred outcome, because it was statistically insignificant in the original model adjusted only for age and time since surgery (see Appendix 4).

a EORTC subscale from QLQ‐C30 or CR‐29.

b *Higher* scores for global health status/QoL and functioning subscales indicate *better* health/QoL and functioning.

c *Higher* scores for symptom subscales indicate *worse* symptoms.

d Urinary symptoms include urinary frequency, urinary incontinence and dysuria.

e Bowel symptoms include blood and mucus in stool, stool frequency, abdominal pain, pain in buttocks/anal area/rectum, bloating, flatulence and faecal incontinence.

*** *P* < .001.

** *P* < .01.

* *P* < .05.

The presence of depression/anxiety remained a statistically significant and strong predictor of poorer outcomes across all domains, with the exception of urinary symptoms. Clinically meaningful differences were observed for global health status/QoL, fatigue, and emotional and social functioning. Arthritis, diabetes, and high blood pressure did not remain significantly associated with any outcomes. Lung disease remained statistically significant only in association with poorer global health status/QoL and physical functioning (*P* < .05).

For participants reporting both limiting comorbidities and depression/anxiety, differences in outcome scores were approximately doubled for domains including fatigue, pain, physical, role, and social functioning, with highly clinically significant differences in outcome scores of >20.

## 4 DISCUSSION

This is the first paper to describe the stability of comorbidity prevalence, individual comorbidities, and patient‐reported limitations of comorbidities, and demonstrate their significant associations with poorer QoL, symptoms, and functioning up to 5 years following CRC diagnosis. We demonstrate that it is not the presence of comorbidities per se, but the limitations on daily activities imposed by comorbidities, which has the greatest impact on health and well‐being.

### 4.1 Frequency and prevalence of comorbidity

Our results demonstrate that 27% of CRC survivors (37% of those with comorbidities) report at least 1 comorbidity that limits their daily activities. Ramsey et al, the only other study to investigate self‐reported comorbidity limitation, found similar findings, with 32% reporting currently limiting comorbidities, although their findings relate to longer term (>5 years) CRC survivors.^12^ Our results also add to the growing evidence that 70% to 80% of CRC survivors are living with at least 1 comorbidity.^9^, ^12^, ^18^

Anxiety and depression are increasingly recognised as common following CRC,^17^ yet CREW adds to only a handful of studies to include them in its assessment of comorbidity.^8^, ^18^ Approximately half of individuals stated that their depression/anxiety was not pre‐existing, but was diagnosed after CRC. Despite low response rates for this question (50%), high rates of depression post cancer diagnosis, particularly in CRC, have been demonstrated elsewhere.^33^ The stability in prevalence of depression/anxiety in the 5‐year follow‐up reported here suggests that often, diagnoses may occur within 3 months of a CRC diagnosis. Our findings highlight the importance of screening for mental well‐being and offering appropriate support. This is emphasised by research detailing how significantly fewer CRC survivors actively seek help for psychological problems than for physical concerns.^34^

The frequency of hypertension, arthritis, diabetes, and lung disease is comparable to other studies,^7^, ^18^ and reflect their prevalence in the general population.^35^ Results demonstrating a ≤10% prevalence of angina, myocardial infarction, and heart failure in the CREW cohort are at odds with higher prevalence in other CRC studies, and in the general population.^8^, ^34^ This likely reflects differences in the criteria for assessing conditions, for example as collective “heart disease” or here, as separate conditions.

### 4.2 Association of comorbidities with QoL, functioning, and symptom outcomes

Our data confirm the importance of understanding whether comorbidities are disrupting daily activities, as these can have a greater, negative impact on health and well‐being during recovery from CRC. Even after accounting for all significant comorbidity predictors, patient‐reported limitations of comorbidities prevailed as a strong and significant predictor across all QoL, functioning, and symptom outcomes. The only exception to this was emotional functioning, where the presence of depression/anxiety held prominent significance. Astrup et al also described associations between limitations of comorbidities and reduced QoL and greater symptom experience, although their study was not limited to CRC.^23^ The only other study to describe similar associations with QoL in CRC patients combined patient‐reported and predefined severity scores, meaning that results do not solely reflect patient reports of limitation.^12^ Studies using clinically derived assessments (predefined weighted scales) of comorbidity severity have been inconsistent in demonstrating a link between greater severity and poorer QoL.^19^, ^22^ Weighted severity scores were designed to predict survival outcomes and therefore do not capture the complexity and impact of living with comorbidities.^21^ Research demonstrating associations between performance status of cancer patients and QoL outcomes supports limitation of daily activities as an important influencer of health and well‐being.^16^ Our findings demonstrate that self‐reported limitations of comorbidities have an important and much greater influence on health and well‐being outcomes, compared to comorbidity presence alone. Whilst the presence and clinically defined severity of comorbidities are important, future assessment should include appraisal of how much they disrupt people's lives.

Perhaps unsurprisingly, the strongest effects of having a limiting comorbidity were seen with pain, physical, and role functioning outcomes. Identified associations with pain are supported elsewhere.^36^, ^37^ However, we describe for the first time the persistent association between limiting comorbidities and symptom outcomes up to 5 years post CRC, in particular the association between comorbidities and poorer urinary and bowel symptoms. Similar associations have been described in rectal cancer,^38^ but this is a new finding in CRC. These findings hold significance, as multiple studies report urinary and bowel symptoms as predominant, persistent, and burdensome following CRC treatment.^9^, ^31^

Previous cross‐sectional studies have demonstrated links between depression/anxiety and poorer QoL, fatigue, pain, physical, and emotional functioning in CRC survivors.^17^, ^19^ Our findings support and expand on this previous literature by demonstrating that depression/anxiety is the most significant individual predictor of poorer health and well‐being outcomes (with the exception of urinary symptoms) in CRC survivors for up to 5 years, even after adjusting for the presence of any limiting comorbidities and other individual conditions. Moreover, our findings suggest a double health and well‐being burden of having both depression/anxiety and any limiting comorbidities.

Interestingly, significant associations of arthritis, as the most limiting comorbidity, disappeared for all outcomes after the inclusion of the presence of any limiting comorbidities in the final models, which likely accounted for the health importance of arthritis. This finding suggests that arthritis, by its limiting nature, is associated with prolonged and poor health and well‐being outcomes, supporting its associations with greater pain and poorer physical functioning seen elsewhere.^8^

### 4.3 Study limitations

Previous cancer studies have demonstrated that participants are less likely to have severe comorbidities than nonresponders.^22^ This should be taken into consideration when interpreting results, as it is possible that our findings may underrepresent the true extent and impact of comorbidities. Assessment of EORTC QLQ‐C30, QLQ‐CR29, and comorbidities was not included within questionnaires until 3 months because of burden of data collection close to diagnosis. Participants were asked whether comorbidities were diagnosed prior to their CRC diagnosis at 24 months; as such, responses are liable to recall bias. The list of comorbidities available for self‐report was limited to 12 individual conditions or disease groups and did not encompass all potential comorbid conditions (for example, obesity). A prior diagnosis of cancer was an exclusion criterion, meaning that previous cancer diagnoses could not be included in comorbidity assessment. Falling response rates over follow‐up mean that apparent trends in comorbidities over time need to be interpreted with caution. Any apparent decline in absolute numbers of individuals reporting comorbidities could be because of more unwell individuals withdrawing from the study.

### 4.4 Conclusions and clinical implications

Our findings highlight the importance of identifying patient‐reported presence and limitations of comorbidities, as important health and well‐being predictors both during and beyond CRC treatment. The stability in prevalence and severity of comorbidity suggests that CRC patients at risk of poorer outcomes up to 5 years following a diagnosis can be identified early, and appropriate support put in place. As such, key consideration should be given to optimising comorbidity management before CRC treatment, and clinical follow‐up that incorporates comorbidity assessment, is individualised, and takes place as soon as possible following a CRC diagnosis.

The International Society of Geriatric Oncology recommends geriatrician involvement in the management of cancer patients with comorbidities, and treatment decisions that consider comorbidities.^39^ We propose that targeted interventions and support services, including multiprofessional management and tailored assessment and follow‐up, may aid recovery of health and well‐being.

The ColoREctal Well‐being (CREW) study highlights the importance of including conditions such as musculoskeletal and mood disorders, and patient‐reported limitations, in future clinical and research assessments of comorbidity. The inclusion of self‐reported health status in the assessment of comorbid CRC patients is a recommendation echoed by NICE multimorbidity guidance^40^ and could help to identify CRC patients at risk of reduced health and well‐being.

## CONFLICT OF INTEREST

Professor Deborah Fenlon has received an honorarium for teaching from Roche.

## FUNDING

The ColoREctal Well‐being (CREW) study is funded by Macmillan Cancer Support grant number 3546834.

## ETHICAL APPROVAL

All procedures performed in studies involving human participants were in accordance with the ethical standards of the institutional and/or national research committee and with the 1964 Helsinki Declaration and its later amendments or comparable ethical standards.

## Supporting information

Data S1: **Appendix 1.** Number and severity of limiting comorbidities reported at 3, 15, 24, 36, 48 and 60 months following primary colorectal cancer surgery

Data S2: **Appendix 2.** Prevalence of individual self‐reported comorbidities at 3, 15, 24, 36, 48 and 60 months following primary colorectal cancer surgery, and prevalence of those reported to limit daily activities at 3 months

Data S3: **Appendix 3.** Number of comorbidities reported at 3 months following colorectal cancer surgery according to socio‐demographic, clinical and treatment characteristics

Data S4: **Appendix 4.** Mean differences in EORTC subscale scores over follow‐up between 3 and 60 months following surgery, estimated from multivariable regression models adjusted for age at baseline and time since surgery

## References

[1] GLOBOCAN . Estimated cancer incidence, mortality and prevalence worldwide in 2012. http://globocan.iarc.fr/Pages/fact_sheets_cancer.aspx.

[2] NCI . National Cancer Institute. Cancer Stat Facts: Colon and Rectum Cancer 2016.

[3] ONS . Office for National Statistics. Cancer survival in England: patients diagnosed between 2010 and 2014 and followed up to 2015. Available from: https://www.ons.gov.uk/peoplepopulationandcommunity/healthandsocialcare/conditionsanddiseases/bulletins/cancersurvivalinenglandadultsdiagnosed/2010and2014andfollowedupto2015, 2016.

[4] Parry C , Kent EE , Mariotto AB , Alfano CM , Rowland JH . Cancer survivors: a booming population. Cancer Epidemiol Biomarkers Prev. 2011;20(10):1996‐2005. 21980007 10.1158/1055-9965.EPI-11-0729 PMC3422885

[5] Feinstein AR . The pre‐therapeutic classification of co‐morbidity in chronic disease. J Chronic Dis. 1970;23(7):455‐468. 26309916 10.1016/0021-9681(70)90054-8

[6] Janssen‐Heijnen ML , Houterman S , Lemmens VE , Louwman MW , Coebergh JWW . Age and Co‐morbidity in Cancer Patients: a Population‐Based Approach *Biological Basis of Geriatric Oncology*. Springer; 2005:89‐107. 10.1007/0-387-23962-6_5 15839192

[7] Edwards BK , Noone AM , Mariotto AB , et al. Annual report to the nation on the status of cancer, 1975‐2010, featuring prevalence of comorbidity and impact on survival among persons with lung, colorectal, breast, or prostate cancer. Cancer. 2014;120(9):1290‐1314. 24343171 10.1002/cncr.28509 PMC3999205

[8] Vissers PA , Thong MS , Pouwer F , Zanders MM , Coebergh JW , van de Poll‐Franse LV . The impact of comorbidity on health‐related quality of life among cancer survivors: analyses of data from the PROFILES registry. J Cancer Surviv. 2013;7(4):602‐613. 23918453 10.1007/s11764-013-0299-1

[9] Downing A , Morris EJ , Richards M , et al. Health‐related quality of life after colorectal cancer in England: a patient‐reported outcomes study of individuals 12 to 36 months after diagnosis. J Clin Oncol. 2015;33(6):616‐624. 25559806 10.1200/JCO.2014.56.6539

[10] Michelson H , Bolund C , Brandberg Y . Multiple chronic health problems are negatively associated with health related quality of life (HRQoL) irrespective of age. Qual Life Res. 2000;9(10):1093‐1104. 11401042 10.1023/a:1016654621784

[11] Elliott J , Fallows A , Staetsky L , et al. The health and well‐being of cancer survivors in the UK: findings from a population‐based survey. Br J Cancer. 2011;105(Suppl 1):S11‐S20. 22048028 10.1038/bjc.2011.418 PMC3251954

[12] Ramsey SD , Berry K , Moinpour C , Giedzinska A , Andersen MR . Quality of life in long term survivors of colorectal cancer. Am J Gastroenterol. 2002;97(5):1228‐1234. 12017152 10.1111/j.1572-0241.2002.05694.x

[13] Naik AD , Uy N , Anaya DA , Moye J . The effects of age, education, and treatment on physical, sexual and body concern symptoms among multimorbid, colorectal cancer survivors. J Geriatr Oncol. 2015;6(4):299‐306. 25920578 10.1016/j.jgo.2015.04.001 PMC4859330

[14] Kurtz ME , Kurtz JC , Stommel M , Given CW , Given B . Predictors of depressive symptomatology of geriatric patients with colorectal cancer: a longitudinal view. Support Care Cancer. 2002;10(6):494‐501. 12353129 10.1007/s00520-001-0338-8

[15] Trentham‐Dietz A , Remington PL , Moinpour CM , Hampton JM , Sapp AL , Newcomb PA . Health‐related quality of life in female long‐term colorectal cancer survivors. Oncologist. 2003;8(4):342‐349. 12897331 10.1634/theoncologist.8-4-342

[16] Elliott BA , Renier CM , Haller IV , Elliott TE . Health‐related quality of life (HRQoL) in patients with cancer and other concurrent illnesses. Qual Life Res. 2004;13(2):457‐462. 15085918 10.1023/B:QURE.0000018476.11278.35

[17] Gray NM , Hall SJ , Browne S , et al. Modifiable and fixed factors predicting quality of life in people with colorectal cancer. Br J Cancer. 2011;104(11):1697‐1703. 21559017 10.1038/bjc.2011.155 PMC3111166

[18] Husson O , Mols F , van de Poll‐Franse LV , Thong MS . The course of fatigue and its correlates in colorectal cancer survivors: a prospective cohort study of the PROFILES registry. Support Care Cancer. 2015;23(11):3361‐3371. 26123601 10.1007/s00520-015-2802-x PMC4584107

[19] Hornbrook MC , Wendel CS , Coons SJ , et al. Complications among colorectal cancer survivors: SF‐6D preference‐weighted quality of life scores. Med Care. 2011;49(3):321‐326. 21224741 10.1097/MLR.0b013e31820194c8 PMC3503529

[20] Thong MS , Mols F , Wang XS , Lemmens VE , Smilde TJ , van de Poll‐Franse LV . Quantifying fatigue in (long‐term) colorectal cancer survivors: a study from the population‐based patient reported outcomes following initial treatment and long term evaluation of survivorship registry. Eur J Cancer. 2013;49(8):1957‐1966. 23453750 10.1016/j.ejca.2013.01.012 PMC3676930

[21] Charlson ME , Pompei P , Ales KL , MacKenzie CR . A new method of classifying prognostic comorbidity in longitudinal studies: development and validation. J Chronic Dis. 1987;40(5):373‐383. 3558716 10.1016/0021-9681(87)90171-8

[22] Deshields TL , Potter P , Olsen S , Liu J . The persistence of symptom burden: symptom experience and quality of life of cancer patients across one year. Support Care Cancer. 2014;22(4):1089‐1096. 24292095 10.1007/s00520-013-2049-3 PMC4929053

[23] Astrup GL , Hofso K , Bjordal K , et al. Patient factors and quality of life outcomes differ among four subgroups of oncology patients based on symptom occurrence. Acta Oncol. 2017;56(3):462‐470. 28077018 10.1080/0284186X.2016.1273546

[24] Vissers PA , Thong MS , Pouwer F , Creemers GJ , Slooter GD , van de Poll‐Franse LV . Prospectively measured lifestyle factors and BMI explain differences in health‐related quality of life between colorectal cancer patients with and without comorbid diabetes. Support Care Cancer. 2016;24(6):2591‐2601. 26715295 10.1007/s00520-015-3052-7 PMC4846693

[25] Fenlon D , Richardson A , Addington‐Hall J , et al. A cohort study of the recovery of health and wellbeing following colorectal cancer (CREW study): protocol paper. BMC Health Serv Res. 2012;12(1):90. 22475242 10.1186/1472-6963-12-90 PMC3382420

[26] DCLG. Department for Communities and Local Government . The English index of multiple deprivation (IMD) 2015—guidance. Available from: https://www.gov.uk/government/uploads/system/uploads/attachment_data/file/464430/English_Index_of_Multiple_Deprivation_2015_-_Guidance.pdf. 2015.

[27] De‐loyde KJ , Harrison JD , Durcinoska I , Shepherd HL , Solomon MJ , Young JM . Which information source is best? Concordance between patient report, clinician report and medical records of patient co‐morbidity and adjuvant therapy health information. J Eval Clin Pract. 2015;21(2):339‐346. 25645368 10.1111/jep.12327

[28] Sangha O , Stucki G , Liang MH , Fossel AH , Katz JN . The self‐administered comorbidity questionnaire: a new method to assess comorbidity for clinical and health services research. Arthritis Rheum. 2003;49(2):156‐163. 12687505 10.1002/art.10993

[29] Aaronson NK , Ahmedzai S , Bergman B , et al. The European‐organization‐for‐research‐and‐treatment‐of‐cancer Qlq‐C30—a quality‐of‐life instrument for use in international clinical‐trials in oncology. J Natl Cancer Inst. 1993;85(5):365‐376. 8433390 10.1093/jnci/85.5.365

[30] Whistance RN , Conroy T , Chie W , et al. Clinical and psychometric validation of the EORTC QLQ‐CR29 questionnaire module to assess health‐related quality of life in patients with colorectal cancer. Eur J Cancer. 2009;45(17):3017‐3026. 19765978 10.1016/j.ejca.2009.08.014

[31] Bailey CE , Tran Cao HS , Hu CY , et al. Functional deficits and symptoms of long‐term survivors of colorectal cancer treated by multimodality therapy differ by age at diagnosis. J Gastrointest Surg. 2015;19(1):180‐188. discussio 88 25213581 10.1007/s11605-014-2645-7 PMC4289079

[32] Snyder CF , Blackford AL , Sussman J , et al. Identifying changes in scores on the EORTC‐QLQ‐C30 representing a change in patients' supportive care needs. Qual Life Res. 2015;24(5):1207‐1216. 25398495 10.1007/s11136-014-0853-y PMC4405431

[33] Leach CR , Bellizzi KM , Hurria A , Reeve BB . Is it my cancer or am i just getting older?: impact of cancer on age‐related health conditions of older cancer survivors. Cancer. 2016;122(12):1946‐1953. 27159822 10.1002/cncr.29914

[34] Wieldraaijer T , Duineveld LA , van Asselt KM , et al. Follow‐up of colon cancer patients; causes of distress and need for supportive care: results from the ICARE cohort study. Eur J Surg Oncol. 2017;43(1):118‐125. 27633339 10.1016/j.ejso.2016.08.011

[35] Alonso J , Ferrer M , Gandek B , et al. Health‐related quality of life associated with chronic conditions in eight countries: results from the international quality of life assessment (IQOLA) project. Qual Life Res. 2004;13(2):283‐298. 15085901 10.1023/b:qure.0000018472.46236.05

[36] Walling AM , Weeks JC , Kahn KL , et al. Symptom prevalence in lung and colorectal cancer patients. J Pain Symptom Manage. 2015;49(2):192‐202. 24973624 10.1016/j.jpainsymman.2014.06.003 PMC4277493

[37] Given CW , Given B , Azzouz F , Kozachik S , Stommel M . Predictors of pain and fatigue in the year following diagnosis among elderly cancer patients. J Pain Symptom Manage. 2001;21(6):456‐466. 11397603 10.1016/s0885-3924(01)00284-6

[38] Mols F , Lemmens V , Bosscha K , van den Broek W , Thong MS . Living with the physical and mental consequences of an ostomy: a study among 1‐10‐year rectal cancer survivors from the population‐based PROFILES registry. Psychooncology. 2014;23(9):998‐1004. 24664891 10.1002/pon.3517

[39] Papamichael D , Audisio R , Horiot J‐C , et al. Treatment of the elderly colorectal cancer patient: SIOG expert recommendations. Ann Oncol. 2008;20(1):5‐16. 18922882 10.1093/annonc/mdn532

[40] NICE . Multimorbidity: clinical assessment and management: NICE guideline short version DRAFT for consultation (March 2016) https://www.nice.org.uk/guidance/GID-CGWAVE0704/documents/short-version-of-draft-guideline. 2016.

