# Supplementary material for: Comorbidities are associated with poorer quality of life and functioning and worse symptoms in the 5 years following colorectal cancer surgery: Results from the ColoREctal Well‐being (CREW) cohort study
Source: Psychooncology. 2018 Sep 13;27(10):2427–35. doi: 10.1002/pon.4845 (PMC6221152; doi:10.1002/pon.4845)
Supplement: Supplementary file 1 — Data S1: Appendix 1. Number and severity of limiting comorbidities reported at 3, 15, 24, 36, 48 and 60 months following primary colorectal cancer surgery [file PON-27-2427-s001.docx]

*Appendix 1.* Number and severity of limiting comorbidities reported at 3, 15, 24, 36, 48 and 60 months following primary colorectal cancer surgery

|  | **How severely has the condition limited your activities**  **n(%)** | | | | Total number of limiting comorbidities |
| --- | --- | --- | --- | --- | --- |
|  | 1-2  Mild | 3-5  Moderate | 6-7  Severe | Missing data |  |
| **3 Months** | 69 (25%) | 170 (62%) | 27 (10%) | 7 (3%) | N = 273 |
| **15 Months** | 64 (31%) | 104 (50%) | 26 (12%) | 15 (7%) | N = 209 |
| **24 Months** | 65 (34%) | 101 (52%) | 22 (11%) | 5 (3%) | N = 193 |
| **36 Months** | 51 (31.7%) | 87 (54.0%) | 18 (11.2%) | 5 (3.1%) | N = 161 |
| **48 Months** | 51 (27.6%) | 104 (56.2%) | 25 (13.5%) | 5 (2.7%) | N = 185 |
| **60 Months** | 34 (22.1%) | 94 (61.0%) | 20 (13.0%) | 6 (3.9%) | N = 154 |
