# Supplementary material for: Comorbidities are associated with poorer quality of life and functioning and worse symptoms in the 5 years following colorectal cancer surgery: Results from the ColoREctal Well‐being (CREW) cohort study
Source: Psychooncology. 2018 Sep 13;27(10):2427–35. doi: 10.1002/pon.4845 (PMC6221152; doi:10.1002/pon.4845)
Supplement: Supplementary file 2 — Data S2: Appendix 2. Prevalence of individual self‐reported comorbidities at 3, 15, 24, 36, 48 and 60 months following primary colorectal cancer surgery, and prevalence of those reported to limit daily activities at 3 months [file PON-27-2427-s002.docx]

*Appendix 2.* Prevalence of individual self-reported comorbidities at 3, 15, 24, 36, 48 and 60 months following primary colorectal cancer surgery, and prevalence of those reported to limit daily activities at 3 months

| **Self-reported comorbidity** | **Prevalence of Comorbidity** | | | | | | |
| --- | --- | --- | --- | --- | --- | --- | --- |
|  | **(n= answered question)** | | | | | | |
|  | **3 Months** | | **15 Months** | **24 Months** | **36 Months** | **48 Months** | **60 Months** |
|  | **Prevalence at 3 months** | **Prevalence of those with comorbidity that report it limits daily activities** | **Prevalence at 15 months** | **Prevalence at 24 months** | **Prevalence at 36 months** | **Prevalence at 48 months** | **Prevalence at 60 months** |
|  |  |  |  |  |  |  |  |
| High blood pressure | 259 (43.2%) | 15 (5.8%) | 214 (41.2%) | 184 (38.5%) | 140 (38.1%) | 141 (39.8%) | 134 (43.8%) |
|  | N = 600 |  | N = 519 | N = 478 | N = 367 | N = 354 | N = 306 |
| Arthritis or rheumatism | 186 (32.0%) | 99 (53.2%) | 174 (34.2%) | 155 (32.6%) | 133 (36.9%) | 126 (36.6%) | 105 (34.3%) |
|  | N = 581 |  | N = 509 | N = 476 | N = 360 | N = 344 | N = 306 |
| Depression or anxiety | 100 (18.2%) | 36 (36.0%) | 71 (14.4%) | 66 (14.0%) | 46 (13.3%) | 51 (15.4%) | 40 (13.7%) |
|  | N = 550 |  | N = 494 | N = 470 | N = 345 | N = 332 | N = 292 |
| Diabetes or high blood sugar | 89 (16.2%) | 12 (13.5%) | 82 (16.5%) | 74 (15.7%) | 65 (19.0%) | 59 (18.0%) | 66 (23.1%) |
|  | N = 547 |  | N = 496 | N = 472 | N = 342 | N = 328 | N = 286 |
| Asthma, chronic lung disease, bronchitis or emphysema | 85 (15.7%) | 30 (35.3%) | 70 (14.3%) | 66 (13.9%) | 49 (14.2%) | 49 (14.6%) | 48 (16.4%) |
|  | N = 542 |  | N = 491 | N = 475 | N = 345 | N = 335 | N = 292 |
| Chest pain or angina | 55 (10.2%) | 22 (40.0%) | 40 (8.1%) | 43 (9.1%) | 28 (8.3%) | 23 (6.9%) | 24 (8.2%) |
|  | N = 540 |  | N = 492 | N = 473 | N = 339 | N = 333 | N = 293 |
| Inflammatory bowel disease, colitis or Crohn’s disease | 46 (8.7%) | 16 (34.8%) | 31 (6.4%) | 27 (5.8%) | 17 (5.0%) | 15 (4.6%) | 15 (5.2%) |
|  | N = 527 |  | N = 482 | N = 468 | N = 340 | N = 329 | N = 288 |
| Heart attack or myocardial infarction | 40 (7.5%) | 16 (40%) | 40 (8.2%) | 36 (7.7%) | 22 (6.6%) | 22 (6.6%) | 20 (6.8%) |
|  | N = 533 |  | N = 490 | N = 469 | N = 332 | N = 333 | N = 293 |
| Stroke or brain haemorrhage | 21 (4.0%) | 9 (42.9%) | 11 (2.3%) | 10 (2.2%) | 8 (2.4%) | 11 (3.3%) | 11 (3.8%) |
|  | N =529 |  | N = 485 | N = 465 | N = 335 | N = 330 | N = 289 |
| Heart failure | 21 (4.0%) | 11 (52.4%) | 25 (5.1%) | 21 (4.5%) | 21 (6.1%) | 18 (5.4%) | 13 (4.5%) |
|  | N = 527 |  | N = 487 | N = 468 | N = 342 | N = 335 | N = 290 |
| Liver disease or cirrhosis | 8 (1.5%) | 1 (12.5%) | 13 (2.7%) | 11 (2.3%) | 11 (3.3%) | 7 (2.1%) | 5 (1.7%) |
|  | N = 525 |  | N = 485 | N = 469) | N = 338 | N = 327 | N = 289 |
| Bleeding from stomach ulcers | 3 (0.6%) | 0 (0.0%) | 2 (0.4%) | 4 (0.9%) | 0 (0.0%) | 5 (1.5%) | 3 (1.0%) |
|  | N = 523 |  | N = 487 | N = 469 | N = 338 | N = 329 | N = 287 |
