# Supplementary material for: Comorbidities are associated with poorer quality of life and functioning and worse symptoms in the 5 years following colorectal cancer surgery: Results from the ColoREctal Well‐being (CREW) cohort study
Source: Psychooncology. 2018 Sep 13;27(10):2427–35. doi: 10.1002/pon.4845 (PMC6221152; doi:10.1002/pon.4845)
Supplement: Supplementary file 3 — Data S3: Appendix 3. Number of comorbidities reported at 3 months following colorectal cancer surgery according to socio‐demographic, clinical and treatment characteristics [file PON-27-2427-s003.docx]

*Appendix 3.* Number of comorbidities reported at 3 months following colorectal cancer surgery according to socio-demographic, clinical and treatment characteristics

|  | | **Number of Comorbidities (at 3M)** | | | | | Presence of comorbidities (>1) | Chi Square test comparing presence versus absence of comorbidities: p-value |
| --- | --- | --- | --- | --- | --- | --- | --- | --- |
|  |  | 0 | 1 | 2 | 3+ | Total |  |  |
| Socio-demographic Factors | Age group (years) |  |  |  |  |  |  | < 0.001^t^ |
|  | <50 | 25 (58%) | 10 (23%) | 5 (12%) | 3 (7%) | 43 | 18 (42%) |  |
|  | 51-60 | 32 (35%) | 31 (34%) | 22 (24%) | 7 (8%) | 92 | 60 (65%) |  |
|  | 61-70 | 59 (27%) | 75 (35%) | 48 (22%) | 35 (16%) | 217 | 158 (72.8%) |  |
|  | 71-80 | 28 (18%) | 45 (29%) | 43 (28%) | 38 (25%) | 154 | 126 (81.8%) |  |
|  | >80 | 8 (16%) | 18 (35%) | 15 (29%) | 10 (20%) | 51 | 43 (84.3%) |  |
|  | Gender |  |  |  |  |  |  | 0.458 |
|  | Male | 113 (29%) | 133 (34%) | 87 (22%) | 63 (16%) | 396 | 283 (71.5%) |  |
|  | Female | 70 (27%) | 78 (30%) | 63 (24%) | 52 (20%) | 263 | 193 (73.4%) |  |
|  | Deprivation Index (quintiles) |  |  |  |  |  |  | 0.206^t^ |
|  | 1^st^ (least deprived) | 42 (31%) | 44 (32%) | 32 (23%) | 19 (14%) | 137 | 95 (69.3%) |  |
|  | 2^nd^ | 37 (26%) | 46 (33%) | 35 (25%) | 23 (16%) | 141 | 104 (73.8%) |  |
|  | 3^rd^ | 33 (27%) | 37 (30%) | 30 (25%) | 22 (18%) | 122 | 89 (73.0%) |  |
|  | 4^th^ | 33 (28%) | 49 (41%) | 22 (18%) | 16 (13%) | 120 | 87 (72.5%) |  |
|  | 5^th^ (most deprived) | 32 (25%) | 32 (25%) | 30 (23%) | 34 (27%) | 128 | 96 (75.0%) |  |
|  | Domestic Status |  |  |  |  |  |  | 0.314 |
|  | Married/Living with partner | 128 (29%) | 145 (32%) | 106 (24%) | 68 (15%) | 447 | 319 (71.4%) |  |
|  | Single/Widowed/ Divorced/  Separated | 42 (25%) | 51 (30%) | 39 (23%) | 36 (21%) | 168 | 126 (75.0%) |  |
|  | Employment Status |  |  |  |  |  |  | < 0.001 |
|  | Employed (Employed FT, PT, on unpaid or sick leave) | 76 (43%) | 60 (34%) | 31 (18%) | 9 (5%) | 176 | 100 (56.8%) |  |
|  | Unemployed (Unemployed or disabled does not work) | 4 (2%) | 12 (41%) | 7 (24%) | 6 (21%) | 29 | 25 (86.2%) |  |
|  | Retired | 89 (22%) | 124 (31%) | 106 (26%) | 88 (22%) | 407 | 318 (78.1%) |  |
| Clinical Factors | Tumour site |  |  |  |  |  |  | 0.133 |
|  | Colon | 109 (25%) | 138 (32%) | 107 (25%) | 77 (18%) | 431 | 322 (74.7%) |  |
|  | Rectum | 74 (33%) | 73 (32%) | 42 (19%) | 38 (17%) | 227 | 153 (67.4%) |  |
|  | Dukes’ stage |  |  |  |  |  |  | 0.307^t^ |
|  | Stage A | 30 (29.4%) | 25 (24.5%) | 29 (28.4%) | 18 (17.6%) | 102 | 72 (70.6%) |  |
|  | Stage B | 97 (28%) | 117 (34%) | 66 (19%) | 65 (19%) | 345 | 248 (71.9%) |  |
|  | Stage C1 | 31 (24%) | 41 (31%) | 38 (29%) | 21 (16%) | 131 | 100 (76.3%) |  |
|  | Stage C2 | 22 (31%) | 23 (33%) | 16 (23%) | 9 (13%) | 70 | 48 (68.6%) |  |
|  | Nodal Involvement |  |  |  |  |  |  | 0.594^t^ |
|  | N0 | 120 (28%) | 134 (32%) | 88 (21%) | 81 (19%) | 423 | 303 (71.6%) |  |
|  | N1 | 29 (23%) | 41 (33%) | 34 (27%) | 20 (16%) | 124 | 95 (76.6%) |  |
|  | N2 | 23 (31%) | 25 (34%) | 16 (22%) | 10 (14 %) | 74 | 51 (68.9%) |  |
|  | How cancer was detected |  |  |  |  |  |  | 0.671 |
|  | Screening | 39 (25%) | 55 (36%) | 36 (23%) | 25 (16%) | 155 | 116 (74.8%) |  |
|  | Symptomatic | 130 (29%) | 139 (31%) | 104 (23%) | 83 (18%) | 456 | 326 (71.5%) |  |
|  | Emergency surgery/other | 12 (40%) | 8 (27%) | 5 (17%) | 5 (17%) | 30 | 18 (60.0%) |  |
|  | Family History (of CRC) |  |  |  |  |  |  | 0.235 |
|  | Yes | 31 (40%) | 25 (32%) | 13 (17%) | 9 (12%) | 78 | 47 (60.3%) |  |
|  | No | 93 (28%) | 111 (34%) | 73 (22%) | 50 (15%) | 327 | 234 (71.6%) |  |
| Treatment Factors | Presence of a stoma |  |  |  |  |  |  | 0.665 |
|  | Yes | 69 (30%) | 73 (32%) | 46 (20%) | 39 (17%) | 227 | 158 (69.6%) |  |
|  | No | 113 (27%) | 136 (32%) | 101 (24%) | 74 (18%) | 424 | 311 (73.3%) |  |
|  | Neo-adjuvant treatment |  |  |  |  |  |  | 0.148 |
|  | Yes | 37 (31%) | 43 (36%) | 19 (16%) | 20 (17%) | 119 | 82 (68.9%) |  |
|  | No | 146 (27%) | 168 (31%) | 128 (24%) | 94 (18%) | 536 | 311 (73.3%) |  |
|  | Adjuvant treatment |  |  |  |  |  |  | 0.538 |
|  | Yes | 76 (32%) | 71 (30%) | 59 (25%) | 32 (13%) | 238 | 162 (68.1%) |  |
|  | No | 107 (26%) | 140 (33%) | 90 (21%) | 83 (20%) | 420 | 313 (74.5%) |  |

^t^ Chi² test for trend used for age group, deprivation index, Dukes’ stage, nodal involvement
