# Supplementary material for: Comorbidities are associated with poorer quality of life and functioning and worse symptoms in the 5 years following colorectal cancer surgery: Results from the ColoREctal Well‐being (CREW) cohort study
Source: Psychooncology. 2018 Sep 13;27(10):2427–35. doi: 10.1002/pon.4845 (PMC6221152; doi:10.1002/pon.4845)
Supplement: Supplementary file 4 — Data S4: Appendix 4. Mean differences in EORTC subscale scores over follow‐up between 3 and 60 months following surgery, estimated from multivariable regression models adjusted for age at baseline and time since surgery [file PON-27-2427-s004.docx]

*Appendix 4.*  Mean differences in EORTC subscale scores over follow-up between 3 and 60 months following surgery, estimated from multivariable regression models adjusted for age at baseline and time since surgery

| **Independent Variables** | **Dependent Variables: EORTC subscales** ^1^ | | | | | | | | | |
| --- | --- | --- | --- | --- | --- | --- | --- | --- | --- | --- |
|  | **Global health status / QoL^2^** | **Fatigue^3^** | **Pain^3^** | **Urinary symptoms^3,4^** | **Bowel symptoms^3,5^** | **Physical functioning^2^** | **Role functioning^2^** | **Emotional functioning^2^** | **Cognitive functioning^2^** | **Social functioning^2^** |
| **1) Comorbidity status *(ref: none):*** |  |  |  |  |  |  |  |  |  |  |
| **- Yes, non-limiting comorbidities** | -2.0 | 2.8 | 3.7* | 1.8 | 1.2 | -3.7* | -1.8 | -1.0 | -1.7 | -1.2 |
| **- Yes, limiting comorbidities** | -14.0*** | 19.1*** | 24.3*** | 7.2*** | 7.4*** | -20.9*** | -20.2*** | -10.7*** | -11.8*** | -16.1*** |
| **2) High blood pressure *(ref: no)*** | -3.1^+^ | 2.3 | 4.0** | 0.6 | -1.1^+^ | -4.0** | -2.9^+^ | 0.1 | 0.6 | -1.6 |
| **3) Arthritis/rheumatism *(ref: no)*** | -8.6*** | 10.9*** | 17*** | 4.2*** | 4.9*** | -12.4*** | -13.2*** | -6.8*** | -5.6*** | -11.6*** |
| **4) Depression/anxiety *(ref: no)*** | -13.7*** | 17.6*** | 15.9*** | 5.8*** | 6.7*** | -16.8*** | -15.6*** | -22*** | -13.9*** | -16.1*** |
| **5) Diabetes/high blood sugar *(ref: no)*** | -3.6^+^ | 3.6 | 5.0* | 3.9*** | -0.2 | -6.1** | -2.5 | 1.8 | 1.2 | 1.4 |
| **6) Asthma/chromic lung disease *(ref: no)*** | -7.3** | 8.7** | 7.0** | 1.8 | 2.8* | -11.9*** | -9.3*** | -4.8* | -5.9** | -5.4** |

*** p<0.001; ** p<0.01; * p<0.05

^+^ was statistically significant (p<0.05) in a bivariate model and multivariable model adjusted only for time since surgery, but became insignificant after adjusting for age.

^1^ EORTC subscale from QLQ-C30 or CR-29

^2^ *Higher* scores for global health status/QoL and functioning subscales indicate *better* health/QoL and functioning

^3^ *Higher* scores for symptom subscales indicate *worse* symptoms

^4^ Urinary symptoms include urinary frequency, urinary incontinence and dysuria

^5^ Bowel symptoms include blood and mucus in stool, stool frequency, abdominal pain, pain in buttocks/anal area/rectum, bloating, flatulence and faecal incontinence
